# Efficacy and safety of direct oral anticoagulants in older adults with atrial fibrillation: a prospective single-centre cohort study

**DOI:** 10.1007/s11739-023-03375-9

**Published:** 2023-07-20

**Authors:** Filippo Catalani, Elena Campello, Giuseppina Occhipinti, Alessandro Zorzi, Marta Sartori, Bruno Micael Zanforlini, Alessandro Franchin, Paolo Simioni, Giuseppe Sergi

**Affiliations:** 1 Department of Medicine, Geriatric Unit, University Hospital of Padova, Via Giustiniani 2, 35128 Padua, Italy; 2 General Medicine and Thrombotic and Hemorrhagic Diseases Unit, Department of Medicine, University Hospital of Padova, Padova, Italy

**Keywords:** Heart disease, Atrial fibrillation, DOACs, Bleeding, Thrombosis, Elderly

## Abstract

**Introduction:**

Direct oral anticoagulants (DOACs) are underused in the elderly, regardless the evidence in their favour in this population.

**Methods:**

We prospectively enrolled anticoagulant-naïve patients aged ≥ 75 years who started treatment with DOACs for atrial fibrillation (AF) and stratified them in older adults (aged 75–84 years) and extremely older adults (≥ 85 years). Thrombotic and hemorrhagic events were evaluated for 12 months follow-up.

**Results:**

We enrolled 518 consecutive patients. They were mostly aged 75–84 years (299 patients; 57.7%) vs. ≥ 85 years (219 patients; 42.3%). Extremely older adults showed higher incidence of all the endpoints (systemic cardioembolism [HR 3.25 (95% CI 1.71–6.18)], major bleeding [HR 2.75 (95% CI 1.77–4.27)], and clinically relevant non-major bleeding [HR 2.13 (95% CI 1.17–3.92)]) vs. older adults during the first year after starting anticoagulation. In patients aged ≥ 85 years, no difference in the aforementioned endpoints was found between those receiving on-label vs. off-label DOACs. In the extremely older adults, chronic kidney disease, polypharmacy, use of antipsychotics, and DOAC discontinuation correlated with higher rates of thrombotic events, whereas a history of bleeding, Charlson Index ≥ 6, use of reduced DOAC dose, absence of a caregiver, use of non-steroidal anti-inflammatory drugs (NSAIDs), and HAS-BLED score ≥ 3 were associated with major bleedings.

**Conclusions:**

Naïve patients aged ≥ 85 who started a DOAC for AF are at higher risk of thrombotic and bleeding events compared to those aged 75–84 years in the first year of therapy. History of bleeding, HAS-BLED score ≥ 3 and use of NSAIDs are associated with higher rates of major bleeding.

## Introduction

Atrial fibrillation (AF) is the most common cardiac arrhythmia in adults, with an estimated prevalence of 2–4% [1]. Age is an independent risk factor for AF, which is present in 0.12–0.16% of subjects under 49 years, 3.7–4.2% of those aged 60–70 years, and 10–17% of those aged 80 years and older [2]. Patients with AF generally have a 5-times higher risk of ischemic stroke than non-AF subjects, whereas AF patients older than 85 years have a 12-time higher risk of ischemic stroke compared with non-AF subjects of the same age [3].

Four randomised controlled trials (RCTs) for direct oral anticoagulants (DOACs) shown that dabigatran, rivaroxaban, edoxaban, and apixaban are no less effective than warfarin in preventing stroke and systemic embolism [4–7]. Ruff’s meta-analysis of these RCTs found that DOACs significantly reduce stroke and systemic embolic events by 19% compared with warfarin, mainly driven by a reduction in hemorrhagic stroke (52%) [8]. Even several practice- and claims-based analysis have largely confirmed the overall efficacy and safety of DOACs compared with vitamin K antagonists (VKA) [9, 10].

Registration trials that validated DOACs for the prevention of ischemic stroke in AF have included a relevant number of patients (around 27,000) aged 75 and over, in varying percentages in the different studies. However, prescription rates for DOACs are generally suboptimal among the elderly [11]. They often present comorbidities, such as renal impairment and anaemia, or concomitant non-steroidal anti-inflammatory drugs (NSAIDs) use, which make them frailer and more vulnerable to hemorrhagic events [12]. Age-related physiological changes may explain the increased risk of bleeding: the reduction in lean body mass (LBM), total body water (TBW), liver function (especially linked to the cytochrome P450) and creatinine clearance (Crcl) alter pharmacokinetics and expose the organism to higher plasma levels of the anticoagulant drug [13].

Several sets of guidelines recommend oral anticoagulant therapy in patients with AF aged 75 years and older, but to date there has been no clinical trial specifically directed at patients aged ≥ 85 years, who are scarcely represented in most of the current literature [14].

The present study aims at evaluating the efficacy and safety profile of anticoagulant therapy with DOACs in two cohorts of old patients with AF (75–84 vs. ≥ 85 years), and the differences in efficacy and safety according to different dosage regimens (standard vs. reduced dose and on-label vs. off-label reduced dose) in patients aged 85 years and older.

## Methods

### Study population

We prospectively enrolled consecutive patients with AF aged 75 years and older, who referred to the Thromboembolic Diseases Unit of the Geriatric Department of University Hospital of Padova, between January 2014 and May 2019 to start anticoagulant therapy with DOACs (dabigatran, rivaroxaban, edoxaban, apixaban). Only patients who had never been treated with any oral anticoagulant drug whose CHA_2_DS_2_-VASc score was ≥ 2 for men and ≥ 3 for women were enrolled. Patients with mechanical heart valves, moderate-to-severe mitral stenosis, concomitant antiphospholipid syndrome (APS) or venous thromboembolism (VTE), current or prior DOAC or VKA therapy, and contraindication to anticoagulant therapy were excluded. Enrolled patients were followed up over a period of 12 months or until therapy was discontinued due to death, adverse events, or patient’s choice.

The protocol was approved by the local Institutional Ethical Committee (Ref: 5157/AO/21-AOP2237). The study complied with the ethical standards of the 1964 Declaration of Helsinki and its later amendments. Written informed consent was obtained from all participants or from their next of kin for those with cognitive impairment.

The cohort was stratified according to patients’ age (older adults, aged 75–84 years vs. extremely older adults, aged ≥ 85 years) and DOACs dose (standard dose vs. reduced dose). The dose reduction criteria according to renal function were in conformity with the summary of product characteristics (SmPC) as follows: dabigatran, 110 mg twice daily if CrCl 30–50 mL/min or age ≥ 80 years; rivaroxaban, 15 mg daily if CrCl 15–49 mL/min; edoxaban, 30 mg daily if CrCl 15–50 mL/min; apixaban, 2.5 mg twice daily if CrCl 15–29 mL/min or if two-out-of-three: serum creatinine (Cr) ≥ 1.5 mg/dL, age ≥ 80 years, weight ≤ 60 kg. Finally, patients were distinguished between those receiving the recommended DOAC dose (on-label dose) vs. the non-recommended one (off-label dose), according to the aforementioned SmPC.

### Data collection and follow-up

At the time of the enrolment, socio-demographic data (age, sex, Body Mass Index [BMI], presence of a caregiver, mobility, history of falls in the previous six months), and blood tests (red blood cell count, hemoglobin, platelet count, transaminases, and serum creatinine [for the estimation of the glomerular filtration rate, eGFR]) [15] were collected from all the patients. We also recorded the presence of the following chronic conditions: heart failure at enrolment (clinically defined as the presence of typical symptoms and signs derived from an abnormality of cardiac structure or function, according to the definition provided by the ESC Guidelines in force at the time of the enrolment [16]), systemic arterial hypertension, peripheral vascular disease, prior myocardial infarction, prior cerebrovascular event, diabetes mellitus, chronic kidney disease (CKD), liver dysfunction, gastroesophageal reflux disease (GERD), dementia, and prior bleeding (major and clinically relevant non-major bleeding). The Charlson Comorbidity Index (CCI) [17], the CHA_2_DS_2_-VASc score and the HAS-BLED score were calculated. Finally, the number of patient’s medications was also recorded and polypharmacy was defined as the concomitant use of 5 or more medications [18].

### Study outcomes

The primary outcome of the study was the assessment of the efficacy and safety of DOACs in extremely older adults compared with older adults. The secondary outcome was the evaluation of the efficacy and safety of the different doses (standard vs. reduced and on-label vs. off-label) in the extremely older adults.

Efficacy was determined by evaluating the composite endpoint systemic cardioembolism (i.e. ischemic stroke, myocardial infarction and peripheral arterial embolism). Safety was evaluated through the occurrence of bleeding complications, which were categorized as major and clinically relevant non-major bleedings, according to the ISTH bleeding definitions [19].

Study endpoints were evaluated at 3, 6 and 12 months after the enrolment, during scheduled outpatient visits. Patients were instructed to refer to our Unit or the Emergency Department in case of presentation of symptoms suggestive of systemic cardioembolism or bleeding.

### Statistical analysis

Quantitative variables were analysed with the Kolmogorov–Smirnov test. Variables with a normal distribution were expressed as means and standard deviations (SD), variables with a non-normal distribution as means and minimum/maximum values. Qualitative variables were expressed as absolute frequencies (n) and percentages (%). Patients’ characteristics at enrolment were compared between age groups with a Chi-square test for the categorical variables, a T-test for the independent data, or a Mann–Whitney test for the continuous variables (in accordance with the normal distribution of the continuous variables). The cumulative incidence for the outcomes (systemic cardioembolism and bleedings) during the 12 months follow-up was estimated using the Kaplan‐Meier method and compared by log‐rank test between the two groups of patients. Patients who died were censored at the time of death. Multivariable Cox proportional-hazards regression analysis was performed to determine the associations between clinical outcomes (i.e. systemic cardioembolism or major bleeding) with patients characteristics which resulted significant at a univariate analysis (i.e. age ≥ 85 years, BMI, presence of a caregiver, polypharmacy, DOACs dose, on-label dose, eGFR, dementia, antipsychotic drugs use, NSAIDs use, CHA_2_DS_2_-VASC ≥ 5, Charlson Comorbidity Index ≥ 6, HAS-BLED ≥ 3, history of bleeding and DOAC discontinuation). To control for possible confounders, we conducted a supplementary analysis using case–control matching based on the binary variable age ≥ 85 years with potential confounding covariates (i.e. gender, BMI, eGFR, liver function, presence of caregiver, type of DOAC, DOAC dose, on-label dose, CHA_2_DS_2_-VASC score ≥ 5, Charlson Comorbidity Index ≥ 6, dementia, heart failure). Multivariable Cox regression analysis was then repeated on the case–control-matched datasets and HR provided.

Previous studies have estimated the annual incidence rate of the event of systemic cardioembolism in extremely older adults at around 12% [6, 14]. Therefore, a study based on a sample of around 500 patients (250 patients per group) needs to be run with 80% power in order to show outcome differences (i.e. older vs. extremely older adults), with a bilateral significance level of 5%. Statistical analysis was performed using the Statistical Package for Social Science 28.0 (SPSS, IBM Corp., Armonk, NY). For propensity score adjustment STAT v. 18.0 was used.

## Results

### Characteristics of the study population

We prospectively enrolled 518 consecutive patients starting anticoagulant therapy with DOACs. The baseline characteristics of the study population are shown in Table 1. The mean age was 83 years and they were mostly women (N = 283 patients, 54.6%). At the time of the enrolment, 299 patients (57.7%) were classified as older (aged 75–84) and 219 (42.3%) as extremely older (aged ≥ 85).

**Table 1:** Characteristics of the study population

Variable	Entire cohort (n = 518)	Age 75–84 (n = 299)	Age ≥ 85 (n = 219)	p value
Age (years), mean (min–max)	83 (79–87)	79 (77–82)	88 (86–90)	< 0.001
Women, *n* (%)	283 (54.6)	139 (46.5)	144 (65.8)	< 0.001
BMI (kg/m^2^), mean ± SD	25.6 ± 3.7	26.2 ± 3.5	24.7 ± 3.7	< 0.001
Caregiver, *n* (%)	249 (48.1)	102 (34.1)	147 (67.1)	< 0.001
Mobility, *n* (%)				< 0.001
Bedridden	35 (6.8)	8 (2.7)	27 (12.3)	
Autonomous	308 (59.5)	225 (75.3)	83 (37.9)	
Walking stick	140 (27)	54 (18.1)	86 (39.3)	
Wheelchair	35 (6.8)	12 (4.0)	23 (10.5)	
History of falls, *n* (%)	120 (23.2)	53 (17.7)	67 (30.6)	0.001
Blood test				
Red blood cell count (× 10^9), mean ± SD	4396.0 ± 545.1	4516.1 ± 525.3	4232.0 ± 529.8	< 0.001
Hemoglobin (g/L), mean ± SD	131.0 ± 16.9	134.6 ± 16.6	126.2 ± 16.1	< 0.001
Platelet count (× 10^9), median (IQR)	211.0 (177.0–255.0)	206.0 (177.5–256.0)	214.0 (177.0–252.0)	0.633
GOT (UI/ml), median (IQR)	22.0 (17.0–27.0)	22.5 (18.0–27.0)	21 (16.0–25.0)	0.06
GPT (UI/ml), median (IQR)	17.0 (12.0–23.0)	18.0 (14.0–25.0)	15.0 (11.0–20.0)	< 0.001
Creatinine (mg/dL), median (IQR)	0.90 (0.76–1.06)	0.88 (0.77–1.04)	0.94 (0.76–1.09)	0.210
eGFR, (ml/min), median (IQR)	54.6 (43.8–69.2)	62.3 (50.0–75.2)	45.0 (37.0–55.0)	< 0.001
DOACs, *n* (%)				
Standard dose	141 (27.2)	111 (37.1)	30 (13.7)	< 0.001
Reduced dose	377 (72.8)	188 (62.9)	189 (86.3)	< 0.001
On-label dose	360 (69.5)	193 (64.5)	167 (76.3)	0.005
Off-label dose	158 (30.5)	106 (35.5)	52 (23.7)	0.005
Dabigatran	139 (26.8)	100 (33.4)	39 (17.8)	< 0.001
Rivaroxaban	189 (36.5)	103 (34.4)	86 (39.3)	< 0.001
Edoxaban	56 (10.8)	23 (7.7)	33 (15.1)	< 0.001
Apixaban	134 (25.9)	73 (24.4)	61 (27.9)	< 0.001
History and comorbidities, *n* (%)				
Congestive heart failure	147 (28.4)	67 (22.4)	80 (36.5)	< 0.001
Systemic arterial hypertension	464 (89.6)	270 (90.3)	194 (88.6)	0.562
Peripheral vascular disease	184 (35.5)	102 (34.1)	82 (37.4)	0.458
Prior myocardial infarction	80 (15.4)	45 (15.1)	35 (16.0)	0.806
Prior ischemic stroke or TIA	138 (26.6)	70 (23.4)	68 (31.1)	0.06
Diabetes mellitus	116 (22.4)	70 (23.4)	116 (21.0)	0.525
CKD	28 (5.4)	17 (5.7)	11 (5.0)	0.845
Liver dysfunction	8 (1.5)	5 (1.7)	3 (1.4)	0.998
GERD	101 (19.5)	58 (19.4)	43 (19.6)	1.0
Dementia	111 (21.4)	38 (12.7)	73 (33.3)	< 0.001
Prior major bleeding	113 (21.8)	52 (17.4)	61 (27.9)	0.05
Prior clinically relevant non-major bleeding	35 (6.8)	17 (5.7)	18 (8.2)	0.290
Polypharmacy, *n* (%)	404 (78.1)	232 (77.9)	172 (78.5)	0.914
Scores, *n* (%)				
Charlson Comorbidity Index ≥ 6	262 (50.7)	119 (39.9)	143 (65.3)	< 0.001
CHA_2_DS_2_-VASC ≥ 5	286 (55.2)	150 (50.2)	136 (62.1)	0.007
HAS-BLED ≥ 3	186 (35.9)	110 (36.8)	76 (34.7)	0.644

*BMI* body mass index, *GOT* glutamic oxaloacetic transaminase, *GPT* glutamic pyruvate transaminase, *eGFR* estimated glomerular filtration rate, *DOACs* direct oral anticoagulants, *TIA* transient ischemic attack, *CKD* chronic kidney disease, *GERD* gastroesophageal reflux disease, *CHA*_*2*_*DS*_*2*_*-VASC*: congestive heart failure, systemic arterial hypertension, age ≥ 75 years, diabetes mellitus, prior stroke or transient ischemic attack or thromboembolism, vascular disease, age 65–74 years, sex category (female), prior myocardial infarction; *HAS-BLED*: systemic arterial hypertension, abnormal kidney and liver function, stroke, bleeding, labile INR, elderly, drugs or alcohol

Extremely older adults were more fragile (in terms of caregiver presence, mobility, and history of falls) compared to older adults; they also presented lower red blood cell count and hemoglobin, and worse liver and kidney functions.

Extremely older adults were mostly treated with reduced DOACs dose (86.3%) compared to the older ones (62.9%); p for difference < 0.001. Additionally, extremely older adults were mostly treated with on-label DOACs dose (76.3%) compared to the older ones (64.5%); p for difference < 0.005.

Dabigatran was more often prescribed to the older compared to the extremely older adults (33.4 vs. 17.8%, respectively; p for difference < 0.001), while edoxaban was more often used for the extremely older compared to the older adults (15.1 vs. 7.7%, respectively; p for difference < 0.001). The Charlson Index was ≥ 6 in 65.3% extremely older vs. 39.9% older adults (p for difference < 0.001), whereas CHA_2_DS_2_-VASc was ≥ 5 in 62.1% extremely older vs. 50.2% older adults (p for difference = 0.007).

### Outcomes

#### Primary outcome

Extremely older adults presented a significantly higher cumulative incidence of all the efficacy (i.e. systemic cardioembolism) and safety endpoints (i.e. major and clinically relevant non-major bleeding), as shown in Table 2. Overall, the most frequent site of major bleeding was gastrointestinal (n = 18, 50%), followed by intracranial (n = 9, 25%). Among clinically relevant non-major bleeding, 23 (47.0%) occurred in the gastrointestinal tract and 16 (32.6%) in the genitourinary tract.

**Table 2:** Incidence of the efficacy and safety endpoints in the entire cohort

Endpoint	Age 75–84 (n = 299)	Age ≥ 85 (n = 219)	HR (95%CI)
Systemic cardioembolism *n* (%)	8 (2.67)	22 (10.05)	3.25 (1.71–6.18)
Major bleeding *n* (%)	13 (4.34)	23 (10.5)	2.75 (1.77–4.27)
Clinically relevant non-major bleeding *n* (%)	20 (6.69)	29 (13.24)	2.13 (1.17–3.92)
All-cause death *n* (%)	10 (3.34)	30 (13.7)	4.57 (2.23–10.02)

**Fig. 1:**
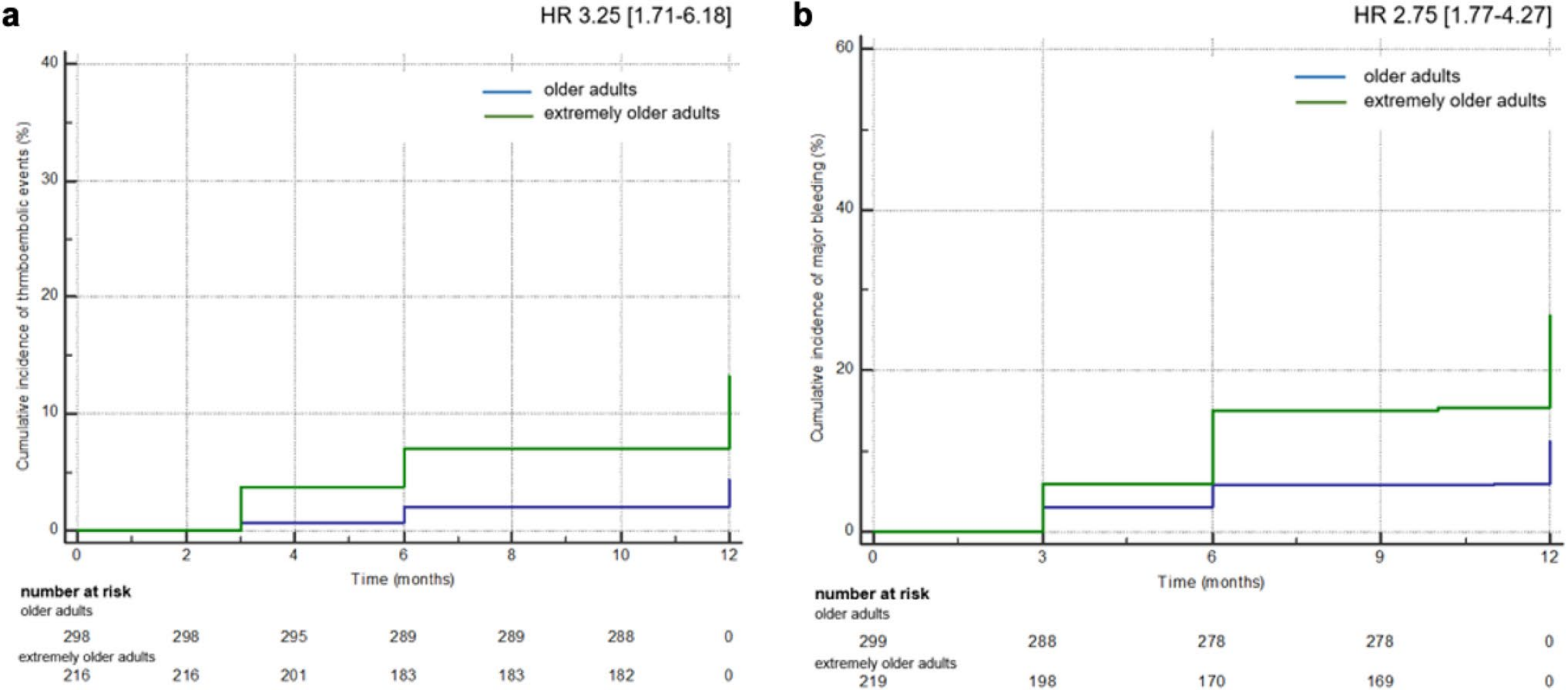
Cumulative incidence of thrombotic events and major bleeding in older and extremely older adults. **A** cumulative incidence and number of patients at risk for thrombotic events, **B** cumulative incidence and number of patients at risk for major bleeding

Considering the cumulative incidence of thrombotic events (i.e. ischemic stroke, myocardial infarction and peripheral arterial embolism) and major bleeding, we confirmed a higher incidence in the extremely older vs. older adults with a HR of 3.25 (95% CI 1.71–6.18) for thrombotic events and 2.75 (95% CI 1.77–4.27) for major bleeding, for 12 months follow-up (Fig. 1). The cumulative incidence of the primary outcomes was confirmed significantly increase in patients older than 85 years vs. those aged 75–84 years even after a case–control matching for possible confounders: HR 2.61 (95% CI 1.04–6.7) for thrombotic events and 1.86 (95% CI 1.07–3.22) for major bleeding, for 12 months follow-up.

#### Secondary outcome

There was no significant difference in any of the considered endpoints for the extremely older adults between patients receiving standard vs. reduced dose and on-label vs. off-label dose both in the first analysis and after case–control matching (Table 3).

**Table 3:** Incidence of efficacy and safety endpoints in extremely older adults, according to standard vs. reduced dose and on-label vs. off-label dose

Endpoint	Standard dose (n = 30)	Reduced dose (n = 189)	HR (95% CI)	On-label dose (n = 167)	Off-label dose (n = 52)	HR (95% CI)
Systemic cardioembolism, *n* (%)	4 (13.3)	18 (9.5)	1.46 (0.46–4.65)	17 (10.1)	5 (9.6)	1.06 (0.37–3.04)
Major bleeding, *n* (%)	3 (10.0)	20 (10.5)	0.94 (0.26–3.37)	16 (9.5)	7 (13.4)	0.68 (0.26–1.76)
Clinically relevant non-major bleeding, *n* (%)	1 (3.3)	28 (14.8)	0.19 (0.02–1.51)	21 (12.5)	8 (15.3)	0.79 (0.33- 1.91)
All-cause death, *n* (%)	4 (13.3)	26 (13.7)	0.96 (0.31–2.99)	24 (14.3)	6 (11.5)	1.28 (0.49–3.34)

#### Risk factors for thrombotic and hemorrhagic events in extremely older adults

Among all the variables, CKD (HR = 2.74 [95% CI 1.07–7.00]), polypharmacy (HR = 1.13 [95% CI 1.01–1.28]), use of antipsychotic drugs (HR = 2.57 [95% CI 1.07–6.16]), and DOAC discontinuation (HR = 3.40 [95% CI 1.45–7.97]) were found to be associated with higher rates of thrombotic events in extremely older adults (Table 4). After case–control matching, only CKD (HR 2.97 [95% CI 1.12–7.9]) and DOAC discontinuation (HR 4.22 [95% CI 1.48–8.7]) remained statistically significant.

**Table 4:** Variables associated with thrombotic events in extremely older adults

Variable	HR	95% CI
CKD	**2.74**	**1.07–7.00**
Polypharmacy	1.13	1.01–1.28
Antipsychotic drugs	2.57	1.07–6.16
Discontinuation of DOAC	**3.40**	**1.45–7.97**

*CKD* chronic kidney disease, *DOAC* direct oral anticoagulant, Polypharmacy was defined as concomitant use of 5 or more medications. Bold indicates variables associated with the outcome after case–control matching analysis

In terms of bleeding risk, a previous bleeding (HR = 2.4 [95% CI 1.56–3.71]), Charlson Index ≥ 6 (HR = 1.14 [95% CI 1.03–1.26]), use of reduced DOAC dose (HR = 2.0 [95% CI 1.07–3.74]), use of rivaroxaban (HR = 2.4 [95% CI 1.56–3.71]), absence of a caregiver (HR = 1.69 [95% CI 1.07–2.68]), use of NSAIDs for more than three consecutive days (HR = 1.76 [95% CI 1.01–3.1]), and high HAS-BLED score (HR = 1.99 [95% CI 1.31–3.02]) were found to be associated with major bleeding in extremely older adults (Table 5). After case–control matching, only previous bleeding (HR 1.77 [95% CI 1.04–3.01]), use of NSAIDs (HR = 2.02 [95% CI 1.01–3.98]), and high HAS-BLED score (HR = 2.0 [95% CI 1.18–3.44]), remained statistically significant.

**Table 5:** Variables associated with major bleeding in extremely older adults

Variable	HR	95%CI
History of bleeding	**2.4**	**1.56–3.71**
Charlson Index ≥ 6	1.14	1.03–1.26
Reduced dose	2.0	1.07–3.74
Rivaroxaban	2.4	1.56–3.71
Off-label dose	1.47	0.99–2.28
Absence of a caregiver	1.69	1.07–2.68
NSAIDs	**1.76**	**1.01–3.1**
HAS-BLED ≥ 3	**1.99**	**1.31–3.02**

*DOAC* direct oral anticoagulant, *NSAIDs* non-steroidal anti-inflammatory drugs (defined as at least once a day for more than three consecutive days); *HAS-BLED*, hypertension, abnormal kidney and liver function, stroke, bleeding, labile international normalized ratio, elderly, drugs or alcohol

Bold indicates variables associated with the outcome after case–control matching analysis

## Discussion

We performed a prospective analysis of a population of 518 old patients affected by AF on anticoagulant therapy with DOACs, composed of a consistent percentage of subjects aged 85 years and older (42.3%).

We found that patients aged ≥ 85 years presented higher rates of all the efficacy and safety endpoints considered, compared to those aged 75–84 years. Hence, of interest are the results recently published by J. Sabbatinelli et al., where age ≥ 80 years was not found to be associated with an increased risk of thrombotic and hemorrhagic events compared to the subjects aged < 80 years [20]. A possible explanation of this apparent inconsistency could be found in the different cut-offs used in the two studies: the five years gap (i.e. 80–85 years) may play a major role, identifying a different subgroup of patients at higher risk in course of anticoagulation, needing to be addressed by future research in the field.

Our real-world data, provide a higher percentage of patients treated with reduced dose of DOACs (72.8% in the overall cohort), compared to the one in most of the DOACs registration trails (5% in the ARISTOTLE, 21% in the ROCKET AF, and 25% in the ENGAGE-AF) [5–7]. This could be explained by the frequent off-label DOAC administration observed in our study. If understanding the underlying reasons of this inappropriate dose prescription falls outside the purpose of our work, we demonstrated that the off-label DOAC use does not provide any advantage in terms of bleeding, consistently with other recent literature on the topic [20].

We found that the use of antipsychotic drugs was associated with higher rates of thrombotic events in extremely older adults. Since possible cardiovascular sequelae could follow the use of these medications (i.e. arrhythmias, diabetes, obesity) [21], the prescribing physician should consider their presence in patients affected by AF on anticoagulant treatment, to comprehensively estimate the overall cardiovascular risk. If the association between CKD and higher rates of thrombotic events has already been demonstrated [22, 23], of interest is the correlation we found between polypharmacy and thrombotic complications among patients aged 85 years and older. This observation may depend on drug-drug interactions and reduced compliance to pharmacological therapy (including anticoagulant therapy), as already postulated elsewhere [24, 25], as well as on the evidence that polypharmacy usually identifies a subset of multi-morbid and inherently frailer patients. Interestingly, presence of CKD and discontinuation of DOACs resulted the only variables associated with cardioembolic events in patients aged ≥ 85 years after additional analysis to minimize the confounding.

The use of reduced DOACs dose was found to be associated with higher rates of major bleeding in patients aged ≥ 85 years: this could be explained by the elderly intrinsically higher bleeding risk [26] and reinforces the European indications, which suggest the dose reduction only when the appropriate criteria are met [1] on the basis of a wide literature on the topic [27, 28]. Rivaroxaban was associated with higher rates of major bleeding in the extremely older patients. This finding was also previously described [29] and could be explained by the possibly consistent percentage of unknown gastrointestinal cancers in such aged population, especially considering that the most frequent site of major bleeding in our cohort was gastrointestinal (50%). However, the additional case–control matched analysis did not confirm this finding after matching for presence of dementia, on-label use of DOACs and Charlson Comorbidity Index ≥ 6, indicating that patients aged ≥ 85 years in this cohort were frailer than those treated with other DOACs.

In patients aged ≥ 85 years, HAS-BLED score ≥ 3 correlated with higher rates of major bleeding, as already shown by other observational studies [30] and several meta-analysis [31–34]. If a high HAS-BLED score should not discourage physicians from starting or continuing an anticoagulant therapy in patients affected by AF, its use could help in identifying those patients at higher bleeding risk. These patients may benefit from an early and more frequent clinical evaluation, in order to reduce/eliminate reversible bleeding risk factors, as suggested by the current guidelines [1]. Consistent with this finding, a history of bleeding and the use of NSAIDs -both items of the HAS-BLED score- were found to be independently associated with major bleeding in patients aged ≥ 85 years [22]. Finally, frailty markers as Charlson Index ≥ 6 and the absence of a caregiver were associated with higher rates of major bleeding in patients aged 85 years and older, as already shown in a geriatric cohort with AF, treated with anticoagulant therapy [35]. Interestingly, history of bleeding, use of NSAIDs, and HAS-BLED ≥ 3 resulted the only variables associated with major bleeding in patients aged ≥ 85 years, after performing additional analysis to minimize the confounding. These results highlight the ability of the HAS-BLED score to characterized the pro-hemorrhagic profile also in extremely old patients.

The strength of the study relies on the enrolment of consecutive patients representing a real-life cohort of anticoagulant-naïve subjects with AF, starting anticoagulation with DOACs and prospectively observed for 12 months for clinical outcomes: such kind of patients are scarcely represented in current literature on the topic. Moreover, the novelty of the study consists in the fact that patients older than 85 years were compared to those aged 75–84 years, hence overall results specifically pertain to the old population. Finally, the multivariable analysis identified the parameters significantly associated with the outcomes of cardioembolism and major bleeding, in patients older than 85 years in therapy with DOACs for AF.

Our study presents some limitations too. Firstly, the small sample size (especially for extremely older patients) brings some inherent limits in the generalization of the results. Secondly, the short follow-up duration prevented us from the observation of any possible long-term event, even though we intended to describe possible sequelae occurring during the first year of follow-up. Thirdly, since death was the most frequent event in our population, the absence of a competing risk analysis maybe led to incorrect estimates of the outcomes. Finally, the observational nature of the study including two unmatched cohort of patients could have produced impaired results for the presence of confounding.

In conclusion, our study shows that anticoagulant-naïve patients aged 85 years and older who started a DOAC for AF are at higher risk of thrombotic and bleeding events compared to those aged 75–84 years during the first year of therapy. Main modifiable drivers for thrombotic events in extremely older patients are the presence of CKD and DOACs discontinuation. History of bleeding, HAS-BLED score ≥ 3 and use of NSAIDs are associated with higher rates of major bleeding. Further studies focused on extremely older adults are needed to better identify higher risk populations, such as those with renal function impairment, and to optimise thromboembolic prophylaxis.
